# The Use of Professionalism Scenarios in the Medical School Interview Process: Faculty and Interviewee Perceptions

**DOI:** 10.3885/meo.2008.Res00249

**Published:** 2008-02-27

**Authors:** James Kleshinski, Constance Shriner, Sadik A. Khuder

**Affiliations:** * Associate Professor of Medicine, Associate Dean for Admissions, The University of Toledo College of Medicine; † Assistant Professor of Family Medicine, Associate Dean for Faculty Development and Curriculum Evaluation, The University of Toledo College of Medicine; ‡ Professor of Medicine and Public Health, Director of Epidemiology, Northwest Ohio Consortium for Public Health, The University of Toledo College of Medicine

## Abstract

**Purpose:**

The purpose of this study was to determine the impact of professionalism scenarios on the medical school admissions process from applicant and faculty perspectives. Specifically, do completing professionalism scenarios as part of the medical school interview process have an impact on both the interviewee's and the faculty's perception of the process and outcome?

**Method:**

Ninety-one faculty interviewed 199 applicants from January 2007 through April 2007 at The University of Toledo College of Medicine. All applicants were asked one standard professionalism scenario in each of their two interviews. A total of six scenarios were used for the entire interviewing season in rotation every two months. A survey was administered by an admissions office staff member to both the interviewed applicants as well as faculty who conducted interviews about how these scenarios impacted their interview experience.

**Results:**

Asking applicants to respond to professionalism scenarios during the interview was described as having a positive influence on their interview experience. This was also associated with leaving an impression on the applicant about what our institution values in its students and contributed an element of personal reflection about what will be expected of them in the medical profession. Applicants more often reported that asking questions about professionalism was an important aspect of the interview than did faculty. Overall, there was an association between the interviewer's perception of the applicant's response and the interviewer's assessment of professionalism.

**Conclusions:**

Professionalism scenarios can be a worthwhile tool for use in the admissions process. The interview process should encourage participation from faculty who value this as an important component in the evaluation of an applicant. Determinants of faculty perception of the role of assessing professionalism in the interview process should be investigated in future research.

One of the many goals of a medical school admissions process is to select and subsequently admit students who are not only academically capable but who will also demonstrate appropriate professional behavior during their medical careers. Several variables may influence an admission committee decision to offer an acceptance to medical school. The objective variables include items such as undergraduate grade point average (GPA) and scores on the Medical College Admission Test (MCAT). Subjective variables include the interview process, letters of recommendation, and response to application essays. One potential pitfall of the admissions process to medical school may be an over-emphasis on the cognitive aspects of an applicant's file relative to the non-cognitive aspects (such as communication/interpersonal skills, social awareness, cultural competency, and professionalism) presented in their application material.^1^ The need to consider non-cognitive aspects is supported in a recent AAMC publication regarding clinical education.^2^ This report noted that competency in professionalism, as well as the ability to engage and communicate with others, are skills “that students have likely begun to develop in experiences prior to medical school. The purpose of the undergraduate medical curriculum is to advance and refine these foundational competencies.”

There has been much published in the literature with respect to assessing professionalism of medical students during their years in medical school.^3^–^6^ Fewer data exist looking at this question at the preadmission level. Eva and colleagues reported on the incorporation of objective structured clinical examination (OSCE) style stations in the medical school interview process at McMaster University.^7^ Kulatunga-Moruzi and colleagues have also looked at non-cognitive assessment in the interview process to medical school using simulated tutorials.^8^ Assessment at this stage of application to medical school may be of significant importance as evidence suggests that exhibiting unprofessional behavior while in medical school is associated with subsequent action by state medical boards.^9^–^10^

To our knowledge, no studies have looked at whether the assessment of professionalism during the medical school interview process influences applicant opinion of the interview or influences the interviewer's ranking of the applicant. The purpose of this study was to determine the impact of professionalism scenarios from applicant and faculty perspectives. Specifically, do completing professionalism scenarios as part of the medical school interview have an impact on both the interviewee and the faculty's perception of the process and outcome?

## Methods

Institutional Review Board approval was obtained to administer a survey to interviewed medical school applicants as well as to medical school faculty who conducted interviews from January 2007 through April 2007 at The University of Toledo College of Medicine. All interviewed medical school applicants to our College of Medicine receive two interviews, both one-on-one in nature.

**Scenario Development and Validation -** The professionalism scenarios were developed by faculty who have clinical experience and patient care responsibilities. Prior to beginning the interview season, two meetings were held with College of Medicine interviewers to obtain their input on the scenarios and recommended changes or different scenarios prior to implementation. From the input of faculty in the various specialties, the original scenarios were modified as suggested. Used in rotation every two months, a total of six scenarios were introduced for the entire interviewing season and are as follows:

1. You come into the hospital one morning during your 3^rd^ year internal medicine clerkship and smell alcohol on the breath of another medical student during hospital rounds on the ward. What issues would you consider important in coming to a decision about what to do?
2. You inadvertently administer the wrong medication to a patient being treated in the hospital. Although the situation is not life threatening, how would you respond to the situation?
3. You are seeing one of your established patients during a routine clinic visit. As you are leaving the room at the end of the visit, the patient asks you out on a date. What issues would you consider important in coming to a decision about what to do?
4. One of your established patients asks you to write her a prescription for an antibiotic. She is not having any symptoms but tells you it's for her brother who doesn't have any health insurance and can't afford the medication. What issues would you consider important in coming to a decision about what to do?
5. Your clinical preceptor (supervisor) asks you to carry out a procedure which you feel is inappropriate or unsafe. How would you handle the situation with your supervisor?
6. One day, a medical school classmate gives you a sheet containing questions for an upcoming exam. How would you handle the situation and what issues would you consider important in coming to a decision about what to do?

**Data Collection Procedures** - As part of the interview experience, all interviewed applicants were asked one standard professionalism scenario in each of their two interviews. The specific professionalism scenario and written instructions were provided to the interviewer by the admissions office along with the applicant's admission packet. The interviewer recorded the applicant response in narrative form on the interviewer rating form, which was returned to the admissions office. The interviewer was also given the flexibility of asking follow-up questions about the scenarios and was instructed to include the applicant's response in narrative fashion as well. In addition to available space for narrative comments, the interviewer rating form also had questions including “Overall assessment of professionalism” and “Final ranking based on interview and application materials” for which the interviewer provides a numeric ranking (0- reject, 1- borderline, 2- accept, 3- high accept, 4- recruit). The interviewer rating form is a standard component of the admissions process and is reviewed by the admissions committee when considering an offer of acceptance for each interviewed applicant.

At the end of the interview day, the applicant was asked to check out at the admissions office. At that time, an admissions office staff member administered the survey to the applicant. A designated drop box was available for the applicant to place the survey where all responses were collected. A description of the purpose of the survey was placed at the top of the questionnaire. The description stated that the survey was entirely voluntary and anonymous and in no way would impact an admissions decision to the College of Medicine. No personal identifiers such as name, phone number, social security number or AMCAS ID were used. The questions for the applicant survey are described in Appendix 1.

After the interview and in addition to completing the interviewer rating form, the faculty interviewer was asked to complete a survey regarding the applicant's response to the professionalism scenario. No information was obtained that would identify the interviewer or the candidate interviewed. The questions for the interviewer survey are shown in Appendix 2. If the interviewer had already completed the survey at least once before, they did not answer questions 5 and 6 again.

**Data Analysis -** Data were analyzed to determine response patterns for both groups as well as differences between applicant and faculty interviewer responses. Association among the responses for both the applicant and faculty interviewer surveys was tested using chi-square analysis and the strength of the association measured using the gamma statistic. The Cochran-Armitage trend test was conducted to identify differences in ranking for the question common to both surveys, “Questions regarding professionalism/ethics are important to ask as part of the interview process”. All analyses were carried out using SAS Version 9.1.

## Results

**Interviewee Survey** - For the 199 interviewed applicants during this time frame, 107 completed the survey (53.7%). Applicant survey responses are summarized in Table 1. For question 1 (overall interview experience), 103 of 107 responded with 76 applicants (73.8%) ranking their overall feeling about the interview experience positively in the 4–5 category. All 107 interviewed applicants who completed the survey responded to the remaining questions. The most frequent response for question 2 (what our institution values) and question 3 (personal reflection) was in the “Agree” category with 54 (50.5%) and 46 (42.9%) respectively. The most frequent answer for question 4 (professionalism questions are important to ask) was in the “Strongly Agree” category with 51 responses (47.7%). A favorable response to question 1 was associated with more favorable responses to the remaining questions. There was strong correlation between responses for question 1 and question 2 (p = .0001, gamma=.74), question 1 and question 3 (p = .0001, gamma=.62), and question 1 and question 4 (p=.0001, gamma=.56).

**Table 1:** Interviewed applicant survey responses by number and percentage

Question	Negatively Influenced		Neutral		Positively Influenced
	**1**	**2**	**3**	**4**	**5**
1. By asking me to respond to the professionalism/ethics scenarios during the interview, my overall feeling about the interview experience was	0 (0)	2 (1.9)	25 (24.3)	43 (41.8)	33 (32.0)
**Question**	**Strongly Agree**	**Agree**	**Neutral**	**Disagree**	**Strongly Disagree**
2. The professionalism/ethics scenarios influenced my impression on what the University of Toledo College of Medicine values in its student body	27 (25.2)	54 (50.5)	22 (20.6)	4 (3.7)	0 (0)
3. Asking the professionalism/ethics scenarios resulted in personal reflection about what will be expected of me as a physician	42 (39.3)	46 (42.9)	17 (15.9)	2 (1.9)	0 (0)
4. Questions regarding professionalism/ethics are important to ask as part of the interview process	51 (47.7)	43 (40.2)	10 (9.3)	3 (2.8)	0 (0)

Of particular note, while 25 applicants gave a neutral ranking for question 1, four applicants strongly agreed and 11 agreed that asking the professionalism scenarios resulted in personal reflection about what will be expected of them as a physician for question 3. As to question 4 for these 25 applicants, 7 strongly agreed and 10 agreed that questions regarding professionalism were important to ask as part of the interview process.

**Interviewer Survey** - Ninety-one faculty conducted 398 interviews. For the 398 faculty interviews, 254 interviewer surveys (63.8%) were completed. Frequencies of interviewer responses to each item are displayed in Table 2. A favorable response to question 1 (overall impression of applicant) was associated with more favorable responses to question 2 (overall assessment of professionalism) and question 3 (final ranking). There was strong correlation between responses for question 1 and question 2 (p = .0001, gamma=.71). Seventy-three of the 84 interviewers (87%) that gave a 4–5 ranking for question 1, also gave a “Strongly Agree” or “Agree” ranking for question 2. Of the 11 interviewers for question 1 that ranked the applicants’ response to the professionalism scenarios as having a negative influence, 7 strongly agreed or agreed that it impacted their rating for question 2.

**Table 2:** Faculty interviewer survey responses by number and percentage

Question	Negatively Influenced		Neutral		Positively Influenced
	**1**	**2**	**3**	**4**	**5**
1. The applicant's response to the professionalism/ethics scenario influenced my overall impression of the applicant	1 (.4)	10 (4.1)	151 (61.4)	68 (27.6)	16 (6.5)
**Question**	**Strongly Agree**	**Agree**	**Neutral**	**Disagree**	**Strongly Disagree**
2. The applicant's response to the scenario impacted my rating to Question 9 on the interviewer rating form- “Overall assessment of professionalism”	15 (5.9)	112 (44.3)	121 (47.8)	3 (1.2)	2 (.8)
3. The applicant's response to the scenario impacted my rating to the question on the interviewer rating form- “Final ranking based on interview and application materials”	4 (1.6)	82 (32.4)	144 (56.9)	21 (8.3)	2 (.8)
4. I believe the applicant's response to the scenario is reflective of how they would conduct themselves as physicians	23 (9.1)	130 (51.6)	75 (29.8)	21 (8.3)	3 (1.2)
5. Questions regarding professionalism/ethics are important to ask as part of the interview process	26 (20.9)	59 (47.6)	27 (21.8)	10 (8.1)	2 (1.6)

A strong correlation between responses for question 1 and question 3 (p=.0001, gamma=.75) was also found. However, 21 interviewers who ranked the applicant's response as having a positive influence (4–5 rating) for question 1 gave a “Neutral” ranking for question 3. Of the 11 interviewers for question 1 that ranked the applicant's response as having a negative influence, 5 strongly agreed or agreed that it impacted their final ranking of the applicant and 6 were neutral. Fifty-one interviewers (40.2%) who put “Strongly Agree” or “Agree” for question 2 responded “Neutral” for question 3.

Also, there was a statistically significant correlation between responses to question 4 (reflective of how applicant would practice) and question 5 (professionalism questions are important to ask) (p=.012) with the gamma value being lower than for other questions (gamma=.41). Sixty interviewers who put “Strongly Agree” or “Agree” for question 4 also gave a “Strongly Agree” or “Agree” ranking on for question 5. Eleven interviewers who responded “Strongly Agree” or “Agree” to question 4 put “Neutral” for question 5. Five interviewers who responded “Strongly Agree” or “Agree” to question 4 put “Disagree” or “Strongly Disagree” for question 5.

**Group Comparison** - One common question was used in the surveys for both applicants and interviewers, “Questions regarding professionalism/ethics are important to ask as part of the interview process.” There was significant difference in the responses to the common question (p=.0001) with the applicants ranking it more positively than faculty.

## Discussion

The findings show that asking medical school applicants to respond to professionalism scenarios during the interview was described as having a positive influence on their interview experience. This was also associated with leaving an impression on the applicants about what our institution values in its students and contributed an element of personal reflection about what will be expected of them in the medical profession. In addition, applicants felt that asking questions about professionalism was an important aspect of the interview process. Even the majority of students who were neutral with respect to the influence the professionalism scenarios had on their interview experience still reported that it resulted in personal reflection about expectations of the profession and that it was important to ask as part of the interview process. While much of the focus on teaching professionalism and ethics occurs during medical school, perhaps simply promoting thoughts about professionalism in applicants at this stage may be worthy first steps as an introduction to the practice of professional and ethical behavior.

For faculty interviewers the findings showed that, overall, there was correlation between the applicant's response to the professionalism scenarios and an impact on the interviewer's assessment of professionalism. However, there were a number of faculty interviewers who, despite marking the applicant's response to the scenario as having a positive influence, were neutral with respect to the final ranking of the applicant based on interview and application materials. Also, less than half of the interviewers who marked the applicant's response to the scenarios as having a negative influence strongly agreed or agreed that it impacted their final ranking; 40.2% of interviewers who strongly agreed or agreed that it had an impact on their overall assessment of professionalism were neutral on the question of final ranking. In these instances, perhaps the interviewer looked at the other factors of the application, including GPA and MCAT, that overrode the issue of professionalism when it came to a final ranking of the applicant. One comment made on question 6 of the interviewer survey included, “On this student, it did not influence me because it wasn't amazingly good or bad; but, some students answer really good or really bad and that does influence my rankings.”

One of the surprising findings was in the responses to the question common to both the applicant and interviewer survey about whether professionalism questions were important to ask as part of the interview process. More applicants ranked this as important than did faculty interviewers. It is understood that the selection of future doctors to medical school is only one piece of the puzzle for maintaining professionalism in the medical profession. Perhaps the less favorable responses by faculty interviewers reflect the ‘hidden curriculum’: that students’ impressions are formed by the interactions they see from faculty everyday in the hallway and hospital wards as opposed to what they are taught via the formal curriculum.^11^–^12^ One consideration may be that the interview process should encourage participation from those faculty who value this as an important component in the evaluation of an applicant.

Our findings need to be interpreted considering the limitations of the data. First, only 53.7% of the interviewed applicants responded to the survey and we cannot rule out the possibility of a non-response bias. Though there was no information that could have linked an interviewed applicant to their completed survey, perhaps one explanation for this low response rate may be interviewee concerns that returning an unfavorable survey could have an unfavorable impact on the admissions committee vote. It may also be the case that non-responders’ decision not to take the time to complete the survey is related to specific characteristics of this subset of applicants.

Also, over time, interviewed applicants may have been made aware of being asked specific scenarios concerning this topic through individuals from their undergraduate institution or sources on the web. To avoid this, the scenarios were used in rotation, with new ones being introduced throughout the interviewing season. There could also have been some variability in how the interviewers asked or addressed the scenarios. To help minimize this, two meetings were held to explain the process and every interviewer received written instructions about the professionalism scenario with each applicant file prior to the interview day.

It would have been optimal to have matched up the applicant feedback to the interviewing faculty feedback to examine any association between them. This was considered during preparation of the study protocol but dropped due to concerns that if the applicant perceived that their response to the survey was being linked to their interviewer, it might alter their response pattern or perhaps lead to a lower response rate among them. In this study, we did not identify a ‘quality’ rating of each scenario, though this would have been useful, as some scenarios may be better than other scenarios. Additionally, it is possible that the weaker results about the influence of the process on the interviewers were related to interviewer feelings about the scenarios. Finally, all survey questions were framed as positive responses; the inclusion of negative questions may have reduced the bias toward more favorable responses.

Despite these limitations, we believe this study provides new information on the impact of asking professionalism scenarios from both the applicant and faculty interviewer perspective, demonstrating that they can be worthwhile in the admissions process. While there are a number of potential ways that an admissions interview process could evaluate facets of professionalism, it is but one aspect of a multi-pronged approach.^13^ In conjunction with formal instruction during medical school in the classroom and at the bedside, as well as attempts to change the culture of an institution leading to a paradigm shift in exposing the ‘hidden curriculum’, the medical profession can address those factors that impede the highest standard of professionalism being realized.^14^ Future studies should consider follow up for students with respect to recall of their medical school interview, professionalism scenarios, and any impressions that they now have after matriculating to medical school. More studies are needed to determine other interview strategies in the admissions process that help identify future medical students’ capacity for professional behavior. Finally, determinants of faculty perception of the role of assessing professionalism in the interview process should be investigated in future research.

## Appendix 1: Interviewed applicant survey for the College of Medicine

**Table:**

**1. By asking me to respond to the professionalism/ethics scenarios during the interview, my overall feeling about the interview experience was:**				
1	2	3	4	5
Negative Influenced		Neutral		Positively Influenced
**2. The professionalism/ethics scenarios influenced my impression on what the University of Toledo College of Medicine values in its student body:**				
Strongly Agree	Agree	Neutral	Disagree	Strongly Disagree
**3. Asking the professionalism/ethics scenarios resulted in personal reflection about what will be expected of me as a physician:**				
Strongly Agree	Agree	Neutral	Disagree	Strongly Disagree
**4. Questions regarding professionalism/ethics are important to ask as part of the interview process:**				
Strongly Agree	Agree	Neutral	Disagree	Strongly Disagree

## Appendix 2: Interviewer survey for the College of Medicine

**Table:**

**1. The applicant's response to the professionalism/ethics scenario influenced my overall impression of the applicant:**				
1	2	3	4	5
Negative Influenced		Neutral		Positively Influenced
**2. The applicant's response to the scenario impacted my rating to Question 9 on the interviewer rating form- “Overall assessment of professionalism”:**				
Strongly Agree	Agree	Neutral	Disagree	Strongly Disagree
**3. The applicant's response to the scenario impacted my rating to the question on the interviewer rating form- “Final ranking based on interview and application materials”:**				
Strongly Agree	Agree	Neutral	Disagree	Strongly Disagree
**4. I believe the applicant's response to the scenario is reflective of how they would conduct themselves as physicians:**				
Strongly Agree	Agree	Neutral	Disagree	Strongly Disagree
*If you have completed this survey before, please stop here. If this is your first time completing the survey, please answer the following questions:*				
**5. Questions regarding professionalism/ethics are important to ask as part of the interview process:**				
Strongly Agree	Agree	Neutral	Disagree	Strongly Disagree
**6. Do you have any suggestions for other ways to assess professionalism/ethics in our applicant pool?**				
